# Design of Polymeric Surfaces as Platforms for Streamlined Cancer Diagnostics in Liquid Biopsies

**DOI:** 10.3390/bios13030400

**Published:** 2023-03-18

**Authors:** Faezeh Ghorbanizamani, Hichem Moulahoum, Emine Guler Celik, Figen Zihnioglu, Tutku Beduk, Tuncay Goksel, Kutsal Turhan, Suna Timur

**Affiliations:** 1 Biochemistry Department, Faculty of Science, Ege University, Bornova, 35100 Izmir, Türkiye; 2 Bioengineering Department, Faculty of Engineering, Ege University, Bornova, 35100 Izmir, Türkiye; 3 EGE SCIENCE PRO Scientific Research Inc., Ege University, IdeEGE Technology Development Zone, Bornova, 35100 Izmir, Türkiye; 4 Silicon Austria Labs GmbH: Sensor Systems, Europastrasse 12, 9524 Villach, Austria; 5 Department of Pulmonary Medicine, Faculty of Medicine, Ege University, Bornova, 35100 Izmir, Türkiye; 6 EGESAM-Ege University Translational Pulmonary Research Center, Bornova, 35100 Izmir, Türkiye; 7 Department of Thoracic Surgery, Faculty of Medicine, Ege University, Bornova, 35100 Izmir, Türkiye; 8 Central Research Testing and Analysis Laboratory Research and Application Center, Ege University, Bornova, 35100 Izmir, Türkiye

**Keywords:** liquid biopsy, biosensor, polymers, cancer, oncology, diagnosis

## Abstract

Minimally invasive approaches for cancer diagnosis are an integral step in the quest to improve cancer survival. Liquid biopsies such as blood samples are matrices explored to extract valuable information about the tumor and its state through various indicators, such as proteins, peptides, tumor DNA, or circulating tumor cells. Although these markers are scarce, making their isolation and detection in complex matrices challenging, the development in polymer chemistry producing interesting structures, including molecularly imprinted polymers, branched polymers, nanopolymer composites, and hybrids, allowed the development of enhanced platforms with impressive performance for liquid biopsies analysis. This review describes the latest advances and developments in polymer synthesis and their application for minimally invasive cancer diagnosis. The polymer structures improve the operational performances of biosensors through various processes, such as increased affinity for enhanced sensitivity, improved binding, and avoidance of non-specific interactions for enhanced specificity. Furthermore, polymer-based materials can be a tremendous help in signal amplification of usually low-concentrated targets in the sample. The pros and cons of these materials, how the synthesis process affects their performance, and the device applications for liquid biopsies diagnosis will be critically reviewed to show the essentiality of this technology in oncology and clinical biomedicine.

## 1. Introduction

A liquid biopsy is a non-invasive diagnostic test involving the analysis of bodily fluid samples (i.e., blood, urine, or cerebrospinal fluid) for the presence of cancer or other abnormal cells. This method can replace traditional biopsy, which involves surgically removing a tissue sample for analysis (known as invasive techniques). Liquid biopsies have the potential to detect cancers, determine their stages, and monitor treatment effectiveness. They may also be used for screening for genetic abnormalities or other pathologic and abnormal conditions [1,2]. Several techniques such as polymerase chain reaction (PCR), next-generation sequencing (NGS), and digital PCR can be used for liquid biopsy analysis. It is important to note that liquid biopsy is still a relatively new technique, and research is ongoing to determine its effectiveness and reliability in clinical settings [3,4]. However, the potential benefits of liquid biopsy, including its noninvasive nature, real-time insights into disease progression, and the ability to inform treatment decisions, make it a promising tool in the diagnosis and management of a range of diseases.

**Figure 1:**
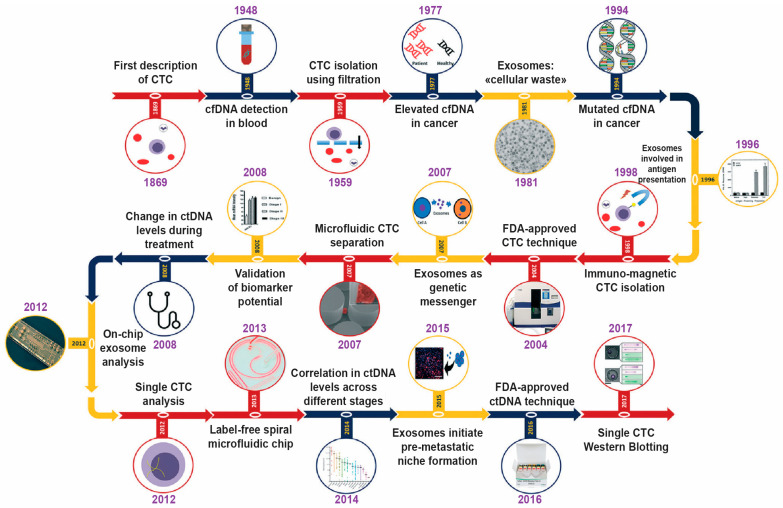
Historical progress of the liquid biopsy research development. The scheme highlights the major milestones in liquid biopsy research, such as EVs, ctDNA, and CTCs. Reused with permission from Ref. [8] ©2019 Royal Society of Chemistry.

One of the primary obstacles in creating effective liquid biopsies is the small amount of cancer-originated materials found in a complex mixture of proteins, cells, and nucleic acids. This can lead to inaccuracies in the detection and analysis of specific biomarkers, including DNA, RNA, and proteins. To address this challenge, different technologies and advanced materials can be utilized, depending on the type of materials shed into the bloodstream by tumors [5]. Tumors regularly release materials in the blood, such as circulating tumor DNA or cells (ctDNA, CTCs) and exosomes (Figure 1). These shed materials, known as tumor-derived extracellular vesicles (EVs), can be detected in a person’s blood or other bodily fluids and can be used as biomarkers for diagnosing and monitoring cancer and other diseases [6].

The development of advanced materials has the potential to revolutionize the performance of liquid biopsy technologies while providing reliable and accurate results. Amongst the various advanced materials that are being developed for use in liquid biopsies include nanoparticles, which are typically made of metals or polymers. Nanoparticles can be customized to capture and isolate specific cells or biomolecules in the body where the functionalization of nanoparticles with specific biomolecules (i.e., antibodies or aptamers) increases their specificity and accuracy [7]. In addition to nanoparticles, polymers including smart polymeric materials have become the subject of interest to improve the sensitivity and specificity of liquid biopsy in order to create personalized treatment plans.

Polymeric materials with easy-to-manipulate process features are suitable for developing different diagnostic tools, including microdevices and biosensors. They are also relatively low-cost compared to other materials, such as metals or ceramics, making them attractive materials in mass-produced devices. In addition, polymers possess a diverse array of physicochemical characteristics that can be adjusted to suit the demands of various uses. Using inspirations from nature and natural polymers to manipulate the chemical structure of polymers can lead to the generation of biocompatible substances suitable for use in medical devices. In addition, the surface of polymeric materials can be manipulated in various ways to enable their use in biological applications. Introducing various functional groups, such as hydroxyl (−OH), carboxyl (−COOH), or amine (−NH_2_) groups, onto the surface creates appropriate interfaces that can interact with biomolecules, such as proteins or nucleic acids, allowing for the specific adsorption or immobilization of these molecules onto their surfaces. Moreover, the surface topography of polymeric materials can be altered through techniques such as micro- and nano-fabrication or natural and synthetic templates. These modifications alter the physical properties of the surface, such as its roughness or wettability, which influence the behavior of cells or other biomolecules that come into contact with the surface.

Given the outstanding features of polymeric materials, this review aims to summarize the latest research on using polymeric micro and nanomaterials in liquid biopsy analysis. It covers the development of nanomaterials for isolation techniques and the use of nano-modified interfaces to improve performance. The review also discusses the application of polymeric materials for electrochemical and fluorescence probes to outline the current state of research in liquid biopsy-based diagnostics and identifies the challenges and future perspectives in this field.

## 2. Application of Polymers in Liquid Biopsy-Based Diagnosis

### 2.1. Non-Fouling Agents

One of the critical challenges in liquid biopsy diagnosis is the potential of biological material accumulation (e.g., such as cells and proteins) on the medical devices used in the procedure. This is known as fouling, which can interfere with the accuracy and reliability of the test results. To address this issue, non-fouling coatings can be applied to the surface of the medical devices used in liquid biopsy analysis. These coatings are designed to minimize the interaction between the device and the biological sample, which helps prevent the accumulation of fouling materials.

**Figure 2:**
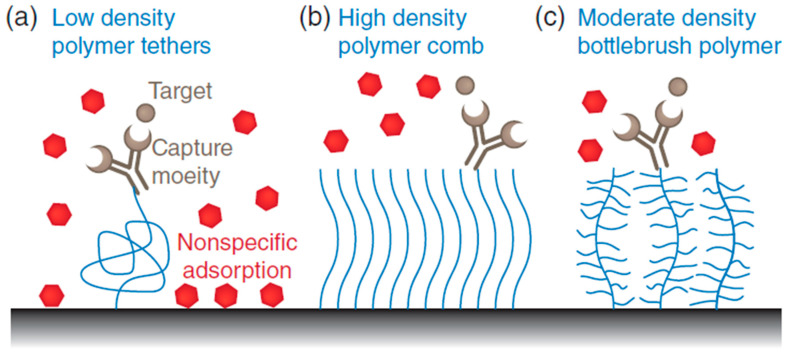
Schematic demonstration of the various surface grafting types of polymers. (**a**) Antibody grafted on a linear polymer and attached to a surface (at low density) enhances flexibility and allows access for capturing target molecules. There is a risk for non-specific attachment of other molecules on the empty gaps (fouling). (**b**) Polymer comb structure made from densely packed linear and hydrophilic polymers to produce antifouling effects. The attachment of targeting molecules will have better specificity. (**c**) Branched bottlebrush polymers are used at lower densities and demonstrate a better antifouling feature than linear polymers.

Polymers are a common choice for non-fouling coatings in liquid biopsy analysis, as they can be easily modified to optimize their performance and tailor them to specific applications. Polyethylene glycol (PEG) is a pioneer antifouling polymer whose ability to inhibit protein adsorption due to its extensive hydrated layer, flexibility, and ability to be functionalized [9]. PEG and some other similar antifouling polymers (e.g., polyvinyl alcohol (PVA)) with suitable surface packing density create a resistant coating layer with great resistance toward non-specific adsorption. Such a layer that omits non-specific adsorption can significantly enhance the selectivity and specificity of the method for its own targeted materials. The antifouling phenomenon is typically achieved via the assistance of several mechanisms including hydration, steric hindrance, ionic solvation, and charge balance [10,11]. The physicochemical properties of the polymers such as the flexibility of the polymer chain, molecular weight, and packing density have direct and critical impact on the antifouling performance of hydrophilic polymers such as PEG chains [12]. This performance of hydrophilic polymer chains is closely related to the formation of a hydrate layer by hydrogen bonds. On the contrary, the implication of charged polymers, such as zwitterionic, results in a strong electrostatic component that positively affects the thickness of the water layer [13]. When using hydrophilic polymer chains as antifouling agents, different surface modification methods such as physical adsorption or covalent grafting can be applicable. The covalently grafted polymer chain or even chemisorbed to the surface can cause a random coil conformation at low density. The increased density makes the random coil state unstable and enlarges the polymer chains to have an arrangement in the “brush regime” [14]. Even though PEG compounds have shown great potential as antifouling agents, their application can be limited by drawbacks such as the vulnerability to oxidative damage and the high molecular weight requirement for creating a stable colloidal state [15]. Other than linear polymers, including PEG (polymer comb), several polymers with different structures, such as brush, dendritic, and hyperbranched polymers, have been utilized as next-generation antifouling coatings that form tightly compact structures required for protein repulsion (Figure 2) [9]. These types of polymers are either zwitterionic polymers or natural-based polymer chains with different morphologies and sizes.

A bottle brush polymer is a type of polymer that involves a main chain (or backbone) that is densely packed with secondary chains attached. These secondary chains are made from monomers that have antifouling properties. A basic example of bottle brush polymers is poly[oligo(ethylene glycol) methyl methacrylate], which has the backbone and the secondary chains made from ethylene glycol units [16]. Other examples of bottle brush polymers include those with backbones made from poly(acrylic acid) [17] or poly(L-lysine) [18], and secondary chains made from poly(2-methyl-2-oxazoline) [19], poly(2-ethyl-2-oxazoline) [18], or poly(N-vinylpyrrolidone) [20].

Dendrimers are a type of polymer with highly organized branching moieties, which gives them a tree-like structure. They are synthesized through a process called divergent synthesis, in which branches are added to a small central molecule in a stepwise fashion [21]. Dendrimers are characterized by their low polydispersity (i.e., uniform size and shape distribution) with a broad application range in liquid biopsy diagnostics. They are often synthesized from an ethylenediamine core [22]. In addition, dendrimers can be created by building inwards (outside-in) via a convergent synthesis process involving attaching branches called “dendrons” to a central structure.

On the other hand, a single-step polycondensation can be used to synthesize hyperbranched polymers with a high density of functional groups [23]. They are commonly used in biosensor applications, and include hyperbranched polyglycerol [24], polyethylene imine [25], polyester [26], and aromatic polyamide [27].

Despite the morphology of the polymer chains, the uniform charge distribution along with the neutral total charge of two oppositely charged moieties on the surface make zwitterionic polymers promising candidates for advanced antifouling-biocompatible materials. Moreover, there are several strategies such as designing new monomers with better hydrophilicity and charge balance, optimizing polymerization conditions, and modifying surface properties by incorporating functional groups that can be used to further enhance the antifouling performance of zwitterionic polymers. On the other hand, natural-based polymers due to their exceptional features including protease resistance, precise control of molecular weight, and broad range of side-chain compositions are regularly utilized in the development of antifouling materials.

While antifouling strategies offer promising solutions to combat fouling, there are several challenges associated with their use. Biocompatibility, biodegradability, and toxicity are important considerations that should be meticulously taken into account, to ensure their safe and effective use.

### 2.2. Isolation and Concentration of Analytes

One of the critical and practical points of liquid biopsy-based diagnosis is the efficient and effective separation of low-concentrated target analytes. Sensitivity and specificity are, therefore, essential performance characteristics of liquid biopsy diagnostics. Polymeric materials can capture different analytes through various mechanisms, depending on the polymer’s specific properties and the target analyte. One common method is affinity-based capture, in which the analyte is specifically attracted to a specific functional group on the polymer. For example, capillary-channeled polymer (C-CP) fibers made of poly(ethylene terephthalate) (PET) were used as a stationary phase in the separation and retrieval of exosomes from various sources, such as urine, buffer, and culture media [28].

Another valuable method for analyte isolation and pre-concentration is using size-based capture, in which the analyte is captured based on its size or shape. This can be accomplished by using a polymer with a porous structure, which allows the analyte to enter the pores and become trapped. Numerous materials have been designed for filtration-based isolation techniques. Polycarbonate-based membranes with pore sizes ranging from 30 to 50 nm are the most commonly used in EV collections [29,30]. Despite the benefits of this method, some significant drawbacks limit their performance. Hence, various contextual cells share comparable features, including the sizes of certain leukocytes and CTCs or the exceedingly large blood cell density that intervene with the efficiency and selectivity of the method. The deformability range is another factor that must be carefully considered in designing microstructures for primary tumor cells and CTCs.

**Figure 3:**
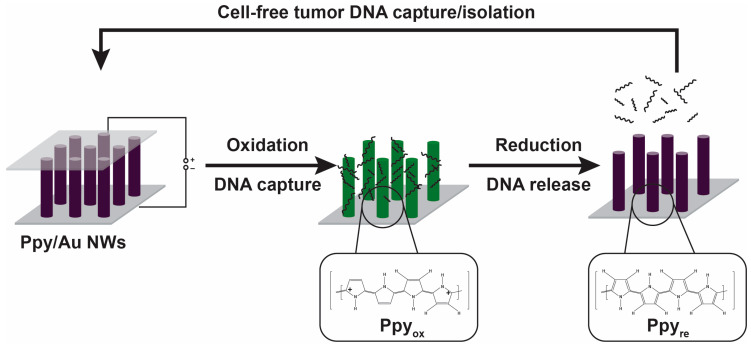
Application of Ppy-coated gold nanowires (Ppy/AuNWs) for the isolation and capture of tumor cfDNA based on the oxidoreduction features under electric fields. Reused with permission from Ref. [36]. ©2016 the authors (open access). Published by Ivyspring International Publisher.

In addition to these mechanisms, polymeric materials can be functionalized with specific ligands or antibodies to capture specific analytes selectively. This can be accomplished through chemical reactions or physical adsorption to attach the ligands or antibodies to the polymer. For example, a polymer with a high surface area and a functional group compatible with the ligand or antibody may be used to capture the analyte via hydrogen bonds or van der Waals forces (noncovalent interactions). Poly(amidoamine) (PAMAM) is a dendrimer frequently used for multivalent recognition and entrapment of CTCs [29,30,31,32,33,34]. The detachment of antibody-decorated PAMAM dendrimers from epithelial cell adhesion molecules (EpCAMs) compared to the antibody alone were found to be 10^6^ times smaller, providing higher capture density and efficiency [33,35]. Jeon et al. developed a new platform using nanostructured conductive polymer polypyrrole (Ppy) to capture and release circulating cell-free DNA (cfDNA) efficiently in unprocessed plasma samples of breast and lung cancer [36]. The developed platform used a polymer-coated gold nanowires structure to trap and isolate cfDNA, followed by the induction of polymer breakage (via an electrical potential) to release the cfDNA (Figure 3).

**Figure 4:**
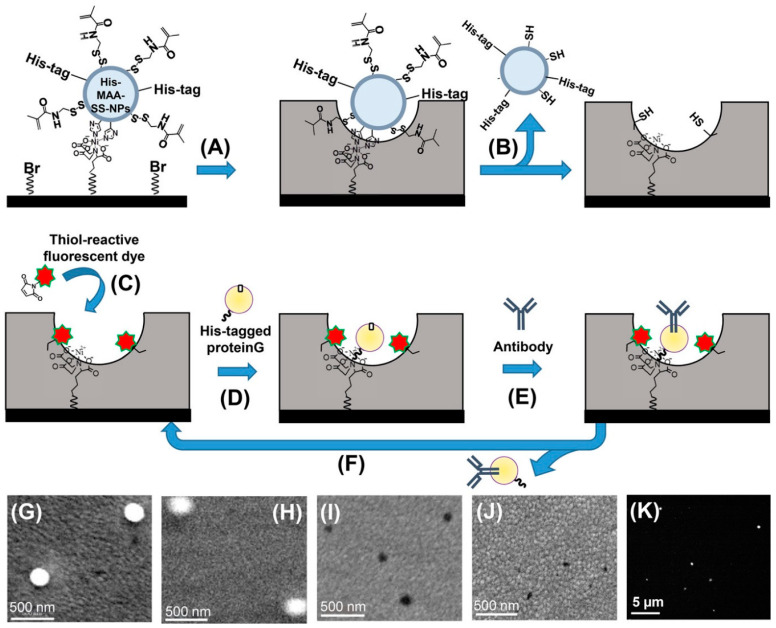
Molecular imprinting of detection cavities conjugated with specific antibodies for detecting intact small extracellular vesicles (sEVs). Nanoprocess of a biocompatible polymer matrix mold creation by immobilization of His-tags over silica nanoparticles (**A**) and their removal (**B**) to create sEV-binding cavities. Post-nanoprocessing via different chemical reactions, including the introduction of fluorescent dyes (**C**), His-tagged protein G (**D**), oriented protein G-based attachment of antibodies (**E**), and regeneration of sEV cavities (**F**). Scanning electron microscopic (**G**–**J**) and fluorescence (**K**) pictures of the different molding steps. Reused with permission from Ref. [37]. ©2020 American Chemical Society.

Molecular imprinting is another promising and fast-growing bio-isolation technique. Using such a method, Takeuchi et al. created EV-shaped cavities by mimicking the structure of conjugating antibodies in polymer matrices where it has been used for the detection of breast cancer by analyzing EVs in tears (Figure 4) [37].

The polymer-based precipitation method in liquid biopsy analysis is a technique that utilizes the ability of certain polymers to bind to specific biomolecules in a liquid sample selectively. This allows the targeted biomolecules to be separated and concentrated from the rest of the sample, enabling their analysis and detection. One example of this method is using magnetic nanoparticles (MNPs) coated with a specific polymer. These MNPs can be added to a liquid sample and then magnetically separated from the rest of the sample using a magnet. The biomolecules of interest will be bound to the polymer-coated MNPs and can be easily separated and purified for further analysis. Riethdorf et al. constructed layer-by-layer magnetic nanospheres (MNs) based on hydrophobic nano-γ-Fe_2_O_3_ coated with poly(styrene/acrylamide) copolymer nanospheres to obtain fast magnetic reaction [38]. Healthy human blood was spiked with breast cancer cells to examine the proposed platform, and the anti-EpCAM-functionalized MNs exhibited high efficiency with 94% capturing capacity in 5 min. The viability of CTCs was approximately 90% and could be transferred to culture, RT-PCR, and immunocytochemistry (ICC) analysis. In another attempt, Liu et al. manufactured a polymer brush and phenylboronic acid combination-based multi-reactive surface to capture and release cancer cells [39]. The polymeric structure can create links with sialic acid (overexpressed in cancer cell membranes) at pH 6.8. Adding glucose and simultaneously increasing the pH to 7.8 could exchange the sialic acid with glucose, creating a stable complex and releasing the caught cells. The created 3D topography could significantly enhance the responsiveness, and accelerate cell capture and the release response rate.

Polymer-based precipitation methods have several advantages over traditional methods, including improved sensitivity and specificity, the ability to analyze various biomolecules, and the non-invasive nature of the technique. However, these methods can also be complex and expensive, and further development and validation are needed before they can be widely used in clinical practice.

Overall, currently conducted research in the liquid biopsy field proves that EVs or ctDNA are preferred indicators over CTCs. Yet, it is important to highlight that possible clinical use of these indicators requires further validation to reach the necessary optimization [40]. The isolation technologies developed for specific markers in complex media have become faster and more efficient than conventional separation techniques such as ultracentrifugation, sedimentation, or density-gradient separation [41].

Recently developed separation techniques could pave the way for liquid biopsy for patients at higher risk and for the cancer markers that are difficult to isolate. Though these techniques provide sufficient sensitivity and selectivity in complex sampling media, cost is still a challenge. Developing the isolation system in a cost-effective manner requires further investigation since the currently developed separation methods are still in research level and far from commercialization [42].

One possible solution to provide further insight into the functionality of the insulation techniques is conducting further clinical investigation. Till date, separation techniques used for liquid biopsy field have been developed in a small scale without a full clinical validation [43]. Thus, more research needs to be conducted to determine the applicability of new methods in terms of throughput and reliability.

### 2.3. Probes for Enhancing the Analyte Detection and the Signal Amplification

Similar to other diagnostic methods, the most critical step is the accurate and sensitive detection of analytes in a liquid biopsy sample. In addition, the ability to amplify the signal produced by detecting an analyte can increase the sensitivity and reliability of the assay, making it possible to detect low levels of analytes in a sample. Several approaches can be used to detect and amplify the signal of analytes in a liquid biopsy sample, including immunoassays, PCR, and microarray analysis. Each of these approaches utilizes specific probes or reagents that bind to and detect target analytes and can be used in combination with signal amplification techniques to increase the sensitivity. Probes can be designed to bind specifically to target analytes or to detect multiple analytes simultaneously using techniques such as microarray analysis, providing a more comprehensive view of the sample. Polymer dots, also known as fluorescent polymer nanoparticles, have emerged as a promising class of probes for liquid biopsy analysis. These nanoparticles can be functionalized with various chemical groups to enable specific binding to target analytes. The nanosized polymer dots can be detected using techniques such as fluorescence microscopy and flow cytometry. Additionally, the high fluorescence of these nanoparticles enables easy visualization and quantification [44,45]. For instance, Balzani et al. synthesized a poly(propylene amine) dendrimer compound with 32 dansyl moieties, highlighting the impact of these dansyl groups to produce strong fluorescence [46].

Fouz et al. prepared a bottlebrush polymer to strengthen the fluorescent signal obtained from a secondary antibody by a 1000-fold and prevent adverse self-quenching effects [47]. The polymer-DNA assembly, in this case, sequesters intercalated fluorophores, preventing their detachment, and can be tethered to the antibody via DNA hybridization. Thus, a fluorescent nanotag can detect target molecules with a bright signal in different techniques, such as confocal fluorescence microscopy, flow cytometry, and dot blots.

In another approach, fluorescently labeled DNA dendrimers were created to strengthen the exosomes’ detection signal [48]. When in contact with exosomes, the aptamers bind to them and release the DNA probe, initiating the hairpin DNA cascade reaction (HDCR) by opening hairpin DNA (HP1) bound to AuNPs. Subsequently, the probe DNA dendrimers link with HP1, leading to an enhanced signal-to-noise ratio. The highly effective method provided an excellent linear response for exosomes derived from HepG2 cells.

To develop a fluorescence liquid biopsy (FLB) analysis technique, Morcuende-Ventura et al. prepared fluorescent dendronized hyperbranched polymers (DHP) created from bis(hydroxymethyl)propionic acid [49]. The output of this study indicated that the inclusion of DHP-bMPA improved the classification performance of FLB in diagnosing pancreatic and ovarian cancers, with a performance reaching 85% (specificity and selectivity) for both pathologies.

Besides the application of polymer dots as imaging agents, several reports show these particles’ potential in different biosensors [50,51]. For example, gold or indium tin oxide (ITO) coated with ferrocene-functionalized PAMAM dendrimers were used in the structure of electrochemical sensors to detect DNA hybridization [52], avidin capture [53], and antibodies [54]. Furthermore, the capture of exosomes on the AuNP surface and the labeling with a polydopamine-coated liquid metal shell-core-core nanohybrid can also generate luminescent signals through electrochemical reactions [55]. In one study, an antifouling electrochemical biosensor based on the conducting polymer poly(3,4-ethylenedioxythiophene) (PEDOT) and a multifunctional peptide was manufactured for CTCs in blood [56]. The designed peptide with antifouling and targeting breast cancer cells capabilities, in addition to the electrodeposited PEDOT, could enhance electron transfer on the detection surface and amplify the signal-to-noise ratio, resulting in an increased sensitivity of the biosensor.

While electrochemical sensors have been utilized to detect DNA, proteins, and small molecules, they are still gaining traction for detecting CTCs or exosomes. There are several reviews available on the use of electrochemical sensors for the detection of various molecules [57,58,59,60]

Surface plasmon resonance (SPR) imaging is one of the methods where the implication of nanoparticles (NPs) could significantly enhance its performance. The NP-enhanced SPR imaging can effectively sense tumor DNA in the patient’s plasma. Bellassai et al. created a dual-functional, low-fouling, and densely packed poly-L-lysine surface layer that prevents non-specific adsorption of blood molecules on the sensor surface [61]. The attachment of peptide nucleic acid probes to this multifunctional polymer layer can complete the circulating DNA sequence, thus facilitating the capture of the analyte from cancer patients. This approach can potentially provide a more accurate and efficient method for detecting tumor DNA in a patient’s plasma.

**Figure 5:**
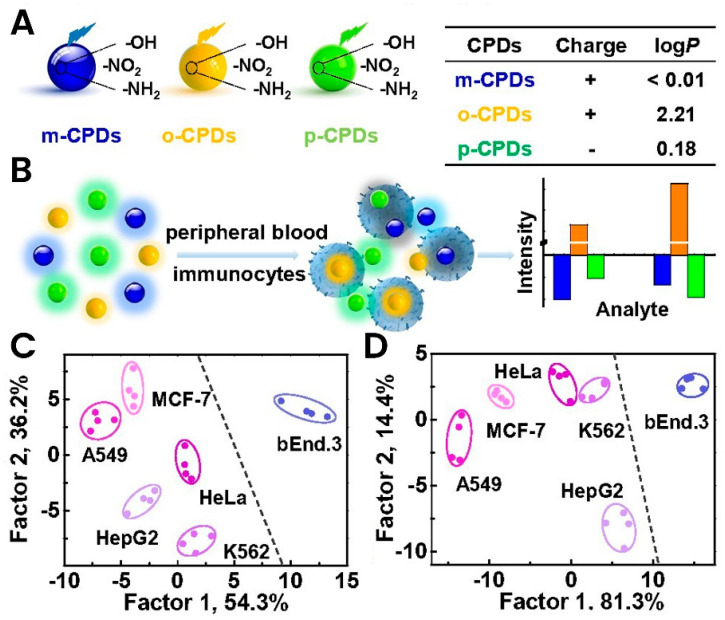
Carbon-based polymer dot (CPD) sensor for cancer diagnosis in the blood showing. (**A**) Schematic demonstration of three CPD forms and their characteristics. (**B**) Demonstration of the luminescence features changes during interaction with target blood cells. (**C**,**D**) Testing of the developed CPDs with various cancer cell lines showing the specific interaction and differentiation of each line. Reused with permission from Ref. [2]. ©2020 Royal Society of Chemistry.

To investigate the potential of polymer dots in the detection of cancer cells, Liu et al. created a sensor for breast cancer diagnosis based on carbon polymer dots (CPDs) in peripheral blood cells [2]. Three polymer structures, including m-Phenylenediamine (mPD), o-phenylenediamine (oPD), and p-phenylenediamine (pPD), were selected for fluorescent synthesis of CPDs and detection of tumor-bearing mice. These CPDs have different surface chemistries, luminescence properties, and functional groups that can be accumulated on the surface of peripheral blood immunocytes. Furthermore, the accumulation of CPDs has led to significant changes in fluorescence intensity due to differences in electronegativity and the proportion of hydrophobic and hydrophilic components on the cell surface. The data collected through linear discriminant analysis (LDA) and hierarchical clustering analysis (HCA) illustrated enhanced precision and strong differentiation of mice with and without tumors (Figure 5).

Following the slight changes in the refractive index (RI) can provide a complete understanding of different biochemical molecules. With this concept, Saha et al. proposed a compact high-index-coated polymer waveguide Bragg grating with a metal undercoat material to develop a sensitive RI-based sensor for detecting EVs in bodily fluids [62]. The developed sensor was assessed using the finite element method (FEM), and coupled-mode theory (CMT) approaches, showing good sensitivity and a wide detection range suitable for EVs analysis. These results show that this approach can be suitable for integration in the real-time, on-chip diagnosis of early-stage cancer.

### 2.4. Microfluidic Technology

Microfluidic technology is a field that involves the manipulation and control of small volumes of fluids, typically in the microliter or nanoliter range, using microscale channels and devices. Microfluidic devices offer several advantages for liquid biopsy applications [63]. First, they can handle small volumes of samples, which is important for non-invasive tests that require only a small amount of fluid. Second, they can provide great sensitivity and specificity, allowing for the accurate detection and analysis of specific biomolecules. Third, microfluidic devices can be designed to perform multiplexed analyses to detect and analyze multiple biomolecules simultaneously. This can be especially useful for cancer diagnosis, providing a more complete picture of cancer characteristics and potential response to treatment.

As mentioned before, microfluidic devices can be used to isolate and purify specific types of biomolecules from complex mixtures. Polymeric materials are useful in developing and enhancing microfluidic technology due to the ease of being molded and patterned using various techniques, such as photolithography, soft lithography, and micro-injection molding. In addition, some polymers can be modified with functional groups or incorporated with nanoparticles to give them specific properties that can be useful in liquid biopsy applications. Therefore, polymeric microfluidic assays have advantages that make them more suitable than non-polymeric assays. These advantages include low cost, ease of fabrication, customizability, flexibility, biocompatibility, and functionality. Taking advantage of polymeric microfluidic assays, Nwankire et al. created an electrochemical lab-on-a-disc (eLoaD) instrument comprising two adhesive layers inserted between three PMMA layers and sputter-coated anti-EpCAM antibodies-functionalized gold electrodes onto the bottom layer [64]. This device allowed computerized quantification of CTCs in the whole blood, which, after applying impedance spectroscopy, can produce comprehensive outputs for the detection and quantification of CTCs in whole blood. In another study, a microfluidic device that utilizes immunoaffinity to separate tumor-derived exosomes directly from plasma was fabricated via soft lithography of polydimethylsiloxane (PDMS) [65]. This device consists of a number of herringbone micromixers, which generate an anisotropic flow to increase the likelihood of exosomes binding to specific antibodies that offer a potential mean of exosome isolation from plasma for further analysis.

A chip made of a thermal-sensitive polymer and graphene oxide (GO) has been created for use in a microfluidic device. It allows for efficient capture and release of CTCs from patients with breast and pancreatic cancer based on immunocapture [66]. A microfluidic chip employing a temperature-sensitive polymer-GO coating has been created to capture and release CTCs from individuals with breast and pancreatic cancer. This composite film allows for temperature-controlled capture and release for the analysis of cells. The film overcomes the limitations of technologies that rely on antibodies attached to the capture surface and can prevent the release of live cells.

Optimizing the integration of functional substrates with microfluidic technology is important for detecting cancer biomarkers. One approach to reducing non-specific adsorption and fluorescence background interference is to coat the substrate with a hydrophilic polymer. Wu et al. proposed a microfluidic device containing zinc oxide nanorods (ZnO NRs) constructed via layer-by-layer electrostatic self-assembly [67]. The introduced polyacrylic acid onto the ZnO NRs (as a hydrophilic layer) significantly suppressed non-specific attachment and provided outstanding antibody immobilization. Under ideal settings, the platform reached 100 fg/mL as LOD for CEA (carcinoembryonic antigen used as a cancer marker). This demonstrates the potential of using hydrophilic polymer coatings on ZnO NR-based microfluidic devices to detect cancer biomarkers.

Polyaniline (PANI) is a well-studied conducting polymer because of its facile synthesis, inherent conductivity, and distinctive doping and de-doping chemistry. The creation of one-dimensional nanostructured PANI has expanded its applications in many fields, including sensors [45].

A PANI nanofiber (NF)-based screen-printed electrode electrochemical sensor that can detect circulating melanoma cells in blood samples with great accuracy has been developed [68]. Furthermore, the microfluidic device showed a significant sensitivity reaching one melanoma cell by milliliter. This demonstrates the potential of using PANI NFs in microfluidic devices for the early detection of cancer. In addition, the small geometry and high surface-to-volume ratio are likely contributing to the enhanced sensitivity of the device.

Combining polymer-based microfluidic devices with microscale magnetic sources has shown promise for various applications. One approach that has gained attention is using magnetic composite polymers created by a polymer matrix doped with magnetic particles or filaments to impart magnetic features [69,70]. Incorporating magnetic materials into polymer-based microfluidic devices is made possible and cost-effective through this method, offering promising potential. However, further research is needed to fully understand and optimize the use of magnetic composite polymers in microfluidic devices and to address any potential challenges or limitations.

Microfluidic platforms are suitable for investigation of small markers in large quantities of raw samples. CTCs, exosomes, and circulating tumor nucleic acids are employed as indicators of diseases at low amounts in the field of non-invasive liquid biopsy research. Several studies have been conducted to validate the potential of microfluidics in biomarker separation [8]. While microfluidic techniques show promise at the research level, commercialization remains a challenge. Polymers used in microfluidic device integration may not be suitable for industrial scale. Moreover, air bubbles and other obstacles during the operation and fabrication process have a significant impact on the performance of microfluidic systems [5].

The current innovative platforms aim to reach cost effectiveness, fast processing time, and less contamination as compared to conventional approaches. Though significant efforts have been made in this regard, most of the microfluidic devices are still not suitable for clinical analysis considering scalability and standardization [71]. The stability and the repeatability of the microfluidics system are crucial during operation. Further research should be conducted to provide more user friendly, cost effective and convenient material selection and microfluidic designs should be considered prior to any comprehensive clinical validation. Knowing that challenges in microfluidic technology exist, FDA-approved liquid biopsy tests are currently limited. Further validation studies involving the specificity, sensitivity, and reproducibility of the microfluidic platforms must be conducted for various tumor types in order to receive more attention from the medical community [72].

## 3. Challenges and Future Perspectives

Polymer-based liquid biopsy methods are diagnostic tests that use small polymer particles to capture and isolate regularly shed materials by tumors into the bloodstream. These methods can be used to detect and monitor cancer and may be useful for identifying treatment options or monitoring the effectiveness of treatment. One of the limitations of polymer-based liquid biopsy analysis methods is the cost and complexity that can vary depending on the specific method used to isolate and detect markers for clinical applications. In general, these methods tend to be more expensive and complex than traditional blood tests, although they may offer benefits related to sensitivity and specificity. Some methods use specialized microfluidic devices or magnetic beads to capture and isolate CTCs or ctDNA, while others use more traditional techniques such as centrifugation or filtration. Another factor influencing the cost and complexity of polymer-based liquid biopsy analysis methods is the type of cancer being tested. Some types of cancer may be more challenging to detect using these methods, which can increase the cost and complexity of the test. For example, certain types of cancer may produce lower levels of CTCs or ctDNA, making them more difficult to detect.

Additionally, the presence of CTCs or ctDNA does not necessarily indicate the presence of cancer, as these substances can also be present in non-cancerous conditions. Therefore, there are several limitations to polymer-based liquid biopsy methods in addition to the cost. Another limitation can be related to the method’s sensitivity and selectivity. Even though polymer-based liquid biopsy analysis methods showed great sensitivity as other diagnostic tests, there is a significant difference in detecting cancer compared to some traditional methods such as tissue biopsy. Tissue biopsy involves obtaining a tissue sample from the tumor itself, which can provide a more direct and accurate cancer diagnosis. There may also be technical limitations to polymer-based liquid biopsy analysis methods, such as requiring specialized equipment or trained personnel to perform the test. Other factors that may influence the outcome of these tests include the patient’s demographic characteristics, such as age and gender, as well as their overall health status and the specific characteristics of the cancer being diagnosed.

Regarding all the mentioned challenges, there is a need for further development and validation of polymer-based liquid biopsy diagnostic methods to improve these tests’ accuracy and reliability and expand their clinical utility. One area of focus for further development is improving the sensitivity and specificity of polymer-based liquid biopsy analysis methods. While these methods have the potential to be highly sensitive and specific, there is still room for improvement in detecting smaller amounts of targets and increasing the accuracy of results. Another area of focus is expanding the range of clinical applications for polymer-based liquid biopsy diagnostic methods. While these methods are already used for various clinical purposes, such as early-stage cancer detection, cancer progression and treatment response monitoring, and the identification of genetic and molecular abnormalities associated with cancer, there is potential for these methods to be used for even more clinical applications. For example, polymer-based liquid biopsy analysis methods may be useful for identifying minimal residual disease (MRD) presence after cancer treatment or monitoring cancer recurrence following treatment. It can also be adapted for non-cancer applications and other pathological abnormalities. Additionally, there is a need for further research to validate the clinical utility of polymer-based liquid biopsy diagnostic methods and to demonstrate their effectiveness in improving patient outcomes. This may involve large-scale clinical trials or other studies to compare the results of polymer-based liquid biopsy diagnostics to other diagnostic tests or to evaluate their impact on treatment decisions and patient outcomes.

## 4. Conclusions

Polymer-based approaches in liquid biopsy have the potential to revolutionize the way that cancer is diagnosed and monitored. These methods offer a non-invasive alternative to tissue biopsy, allowing for the detection and monitoring of cancer using a simple blood test. One of the main potential benefits of polymer-based liquid biopsy methods is the ability to detect cancer at an early stage, when it may be more treatable. These methods may also be useful for cancer progression and therapy response monitoring, helping to identify the most efficient treatment options for individual patients. In addition, polymer-based liquid biopsy analysis methods provide valuable knowledge about cancer genetics and molecular characteristics. This information may be useful for identifying targeted therapies or personalized treatment approaches for individual patients. The potential of polymer-based approaches in liquid biopsy is significant, with the potential to improve cancer diagnosis and management as well as patient outcomes. However, further development and validation of these methods will be necessary to fully realize their potential in clinical practice.

## Data Availability

No new data were created or analyzed in this study. Data sharing is not applicable to this article.
